# Can Cigarette Alternatives Deliver a Safer Fix?

**DOI:** 10.1289/ehp.119-a286

**Published:** 2011-07-01

**Authors:** Cynthia Washam

**Affiliations:** Cynthia Washam writes for *EHP*, *Oncology Times*, and other science and medical publications from South Florida.

Tobacco harm reduction—encouraging the use of cigarette alternatives as a way to reduce the public health impact of smoking—is the subject of fierce debate in the public health community.^1^^,^^2^ Some believe such alternatives perpetuate nicotine addiction in smokers and may even be manufactured and marketed specifically to keep smokers smoking.^3^ “The goal should be relief from addiction to nicotine, not long-term maintenance,” says Norman Edelman, chief medical officer for the American Lung Association.

Others see cigarette alternatives as a way for smokers to cut their health risk. “I saw patients with lung cancer or COPD [chronic obstructive pulmonary disease] who, despite their serious illness, could not quit,” says Brad Rodu, a professor of medicine and Endowed Chair of Tobacco Harm Reduction Research at the University of Louisville. “For smokers who can’t quit, we are obligated as a society to inform them that they have far safer ways to use [tobacco].”^4^

One familiar alternative is smokeless tobacco, which is sold as chewing tobacco or moist snuff (including tiny teabag-like sachets known as snus). A newer alternative is dissolvable tobacco, or finely milled tobacco shaped into pellets, strips, or toothpick-size “sticks” that dissolve in the mouth. Still another option is the battery-operated electronic cigarette, which produces nicotine vapor.

On one point proponents and opponents of these products agree—they’re less harmful than cigarettes. They typically have no more nicotine than cigarettes, and some have far less. However, Scott Tomar, a professor in the University of Florida Department of Community Dentistry and Behavioral Science, points out, “It is not just the amount of nicotine but the route of administration and speed of absorption that determine its physiological effects.” He adds, “It is not the nicotine per se that is the primary harmful substance in tobacco products.”

Perhaps more important, most alternatives contain fewer tobacco-specific *N*-nitrosamines (TSNAs) and other carcinogens than cigarettes because the tobacco is cured differently.^5^ Moreover, much of the harm attributed to cigarettes—for both active and passive smokers—comes from combustion of the tobacco during smoking.^6^

But “not as harmful” is not necessarily the same as “safe,” says Danny McGoldrick, vice president of research for the Washington, DC–based nonprofit Campaign for Tobacco-Free Kids. McGoldrick and many public health researchers are concerned about how few studies have been conducted on the health effects of cigarette alternatives. The exception is smokeless tobacco, which has been listed as a known human carcinogen by the National Toxicology Program since 2000.^7^ The most common side effect of smokeless tobacco use is oral leukoplakia (white lesions on the inside of the mouth).^8^

**Figure:**
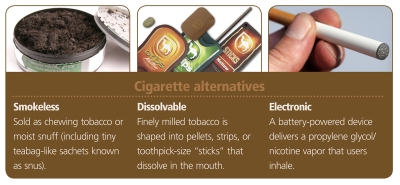
Cigarette alternatives © Left to right: Kris Hanke/iStockphoto; Christina L. Rainey and John V. Goodpaster/Indiana University-Purdue University Indianapolis; Steve Meddle/iStockphoto

A better understanding of the chemical composition of dissolvable tobacco products would open the door to research on oral health effects of using these products. The first published chemical analysis of dissolvable tobacco was published in 2011 by analytical and forensic chemist John Goodpaster and colleagues at Indiana University–Purdue University Indianapolis. His analysis showed that dissolvables contain nicotine levels comparable to those in a single cigarette.

Although the authors did not study health effects of using dissolvable tobacco, they point out that nicotine can be converted into carcinogenic TSNAs in the body, and that nicotine itself can adversely affect gum and tooth health as well as inhibit apoptosis in oral cancer cells.^9^ “Oral cancer is a major [potential] concern,” Goodpaster says.

The analysis also revealed numerous flavor compounds, sweeteners, binders, and humectants. Of the flavor compounds identified, ethyl citrate is acutely toxic with oral dosing; cinnamaldehyde is an oral irritant; and coumarin, a liver and kidney toxicant, has been banned for decades as an additive in foods, although not in tobacco.^9^ Moreover, the fact that dissolvable tobacco is meant to be fully dissolved in the mouth and eventually swallowed “introduces questions in terms of its effect on the gastrointestinal tract,” Goodpaster says.

The e-cigarette departs from tobacco altogether. Instead, users add drops of liquid nicotine to the battery-powered device, which delivers a propylene glycol/nicotine vapor that users inhale. Because e-cigarettes contain no tobacco, they have not been regulated by the Food and Drug Administration (FDA), although a recent court decision changed that.^10^ The FDA expects soon to propose regulation of e-cigarettes under the Family Smoking Prevention and Tobacco Control Act.^11^^,^^12^

A large number of e-cigarettes are imported from China, and “we have no idea of the variation and extent of quality control,” says David Abrams, executive director of the Washington, DC–based Steven A. Schroeder National Institute for Tobacco Research and Policy Studies. A 2009 FDA study of 18 types of e-cigarettes found only trace levels of TSNAs in the devices, comparable to levels found in nicotine patches and nicotine gum and orders of magnitude lower than those found in tobacco cigarettes.^13^

This and other laboratory studies suggest e-cigarettes may be safer than the real thing. However, the FDA study did reveal two problems: wide variation in the amount of nicotine, even in samples of the same product; and in one brand of e-cigarette, the presence of diethylene glycol, a toxic chemical found in antifreeze.^13^

E-cigarette manufacturers are not allowed to claim their products aid in smoking cessation because they have not conducted clinical trials. Yet some researchers believe the devices may in fact prove helpful. “E-cigarettes are a promising strategy in helping people quit,” says Michael Siegel, a Boston University School of Public Health physician and community health professor.

Siegel recently reported on a survey he conducted of smokers who had purchased e-cigarettes for the first time several months earlier.^14^ Of 216 respondents, 66.8% reported cutting down how many cigarettes they smoked, and 31.0% reported quitting cigarettes altogether for at least 6 months. Of those who reported quitting smoking, 34.3% were using no nicotine at all, while 56.7% were still using e-cigarettes.

Siegel and his coauthors pointed out a number of limitations to the study, including the low response rate (4.5%) and the possibility that smokers who had tried but failed to quit would be less likely to complete the survey. Nevertheless, they write, “The finding that most individuals who used e-cigarettes at least reduced the number of tobacco cigarettes they smoked suggests that if proven safe, e-cigarettes may be a potentially important tool for harm reduction.”^14^

But Jonathan Winickoff, a Boston pediatrician and former chair of the American Academy of Pediatrics Tobacco Consortium, worries about the public health impact of seeing an influx of people who appear to be smoking. “When these products are smoked in areas where smoking is prohibited, they may cause former smokers to crave cigarettes,” he says. Antismoking advocates also fear that sweet flavors and easy use make cigarette alternatives particularly alluring to teens. Moist snuff, dissolvable tobacco, and e-cigarettes are all available in different flavors, which the FDA prohibits in regular cigarettes to discourage youth from smoking.^12^

In 2010 the Centers for Disease Control and Prevention reported a nationwide prevalence of smokeless tobacco use of 15% for high-school boys and 2% for high-school girls, with white students using these products the most.^15^ An earlier study indicated teenage boys who used smokeless tobacco were three times as likely as nonusers to be smoking cigarettes four years later.^16^ “If I wanted to get large numbers of people addicted to nicotine,” Winickoff says, “I would probably promote these products.”
